# Utilization of Immediate Postpartum Long Acting Reversible Contraceptives among Women Who Gave Birth in Public Health Facilities in Eastern Ethiopia: A Cross-Sectional Study

**DOI:** 10.1155/2021/1307305

**Published:** 2021-11-10

**Authors:** Ahmedin Aliyi Usso, Hassen Abdi Adem, Yadeta Dessie, Abera Kenay Tura

**Affiliations:** ^1^School of Nursing and Midwifery, College of Health and Medical Sciences, Jijjiga University, Jijjiga, Ethiopia; ^2^School of Public Health, College of Health and Medical Sciences, Haramaya University, Harar, Ethiopia; ^3^School of Nursing and Midwifery, College of Health and Medical Sciences, Haramaya University, Harar, Ethiopia; ^4^Department of Obstetrics and Gynaecology, University Medical Centre Groningen, University of Groningen, Groningen, Netherlands

## Abstract

**Objective:**

Although importance of postpartum family planning is essential and immediate postpartum insertion of long acting and reversible contraceptives (LARC) is recommended, evidence on its uptake and associated factors is limited in Ethiopia. This study was conducted to assess utilization of immediate postpartum LARC among women who gave birth in selected public health facilities in eastern Ethiopia.

**Method:**

An institution-based cross-sectional study was conducted among randomly selected women who gave birth in selected public health facilities in eastern Ethiopia from 10 March to 09 April 2020. At discharge, all eligible women who gave birth in the facilities were interviewed using a pretested structured questionnaire. Data were entered using EpiData 3.1 and analyzed using SPSS 24. Bivariable and multivariable logistic regression analyses were conducted to identify factors associated with utilization of immediate postpartum LARC. Adjusted odds ratio (aOR) with 95% confidence interval was used to report association, and significance was declared at *p* value < 0.05.

**Results:**

From a total of 546 women invited to the study, 530 (97.1%) participated in the study and 98 (18.5%; 95% CI: 15.1%, 22.0%) reported starting long acting reversible contraceptives. Women who reported discussing about contraceptives with partners (aOR = 6.69, 95% CI: 3.54, 12.61) and receiving postpartum counselling on contraceptives (aOR = 5.37, 95% CI: 3.00, 9.63) were more likely to using contraception. However, women who live >30-minute walking distance from the nearest health facility (aOR = 0.47, 95% CI: 0.26, 0.85) and reported disrespect and abuse during childbirth (aOR = 0.22, 95% CI: 0.12, 0.40) were less likely to start LARC.

**Conclusions:**

Almost one in five women delivering in public health facilities in eastern Ethiopia started using LARC. Provision of respectful maternity care including counselling on the importance of immediate postpartum family planning is essential for increasing its uptake.

## 1. Introduction

Contraception is a key life-saving intervention to reduce maternal and neonatal deaths [1, 2]. Globally, universal access to quality contraception decreases unintended pregnancy by 70%, maternal death by 67%, and neonatal death by 77% [1]. Delayed initiation of contraception during postpartum period leads to unintended pregnancy, a significant global public health problem [3]. The studies show that, every year, 41% of total pregnancies (208.2 million) in the world [4] and 40% of total pregnancies (189.6 million) in low and middle income countries (LMIC) [5] while it is around 14 million in sub-Saharan Africa (SSA) [6] were unintended. Similarly, estimated annual burden of unintended pregnancy is also high in Ethiopia: 38% nationally [7], 30% in Oromia region [8], and 27% in rural eastern Ethiopia [9].

In addition, the overall trend of unintended pregnancy was remained unchanged. For example, over the last 20 years, the overall rates of unintended pregnancies (per 1000 women of 15-49 years) decreased by 15% globally (79 in 1990 versus 64 in 2019), by 18% in LMIC (East and Southeast Asia) (60% in 1990 versus to 58% in 1990), and by 12% in sub-Saharan Africa (SSA) (103% in 1990 versus to 92% in 2019) [10] and by 17% in Ethiopia (42% in 2008 versus 25% in 2016) [7].

An unintended pregnancy increases the risks of pregnancy-related complications like maternal and neonatal deaths, abortion, preterm delivery, low birth weight, and postpartum hemorrhage [11]. For instance, unintended pregnancy accounts for 210 maternal deaths per 100,000 annual live births in the world [12], 231 maternal deaths per 1000 annual live births in LMIC [12], 534 maternal deaths per 100,000 annual live births in SSA [12], and 412 maternal deaths per 100,000 annual live births in Ethiopia [7]. Besides, 17 neonatal deaths per 1000 annual live births in the world [13], 27 neonatal deaths per 1000 annual live births in SSA [14], and 92 neonatal deaths per 1000 annual live births in Ethiopia [7] occurred due to unintended pregnancy. Globally, 61% and 25% of unintended pregnancy resulted in premature birth and abortion, respectively [12].

Currently existing worldwide public health recommendation, innovative technology and cost-effective strategy, and policy for fighting unintended pregnancy and its fatal negative effects are increasing the access and coverage of LARC utilization particularly in immediate postpartum, the first 48 hours of child birth [15]. However, the global coverage of LARC use remained unchanged over the last 10 years (6% in 2003 versus 9% in 2012) [11, 16]. Moreover, the level of LARC utilization is below the target in general: around 15% in LMICs [17] and 11% in Ethiopia with a little increment in LARC utilization (9% in 2005 versus 11% in 2019) [18].

Previous studies show factors such as family size, mode of delivery, and fear of side effects [19, 20]; age, religion, marital status, and education of women were associated with LARC methods used [21, 22]. However, there are various dimensions of factors that affect women's utilization of immediate postpartum LARC, but little was known about factors associated with the use of immediate postpartum LARC among women in an immediate postpartum period [23]. Hence, addressing the gaps on utilization and factors associated with immediate postpartum LARC utilization is a top priority to improve its utilization. Despite this, a few studies conducted were focused on hospital and focused on urban and method-specific (IUCD) [21, 22]. Overall, there is limited information on immediate postpartum LARC in Ethiopia in general and East Hararghe Zone in particular. Therefore, this study was conducted to assess utilization of the immediate postpartum LARC and associated factors among women who gave birth in selected public health facilities in East Hararghe Zone, eastern Ethiopia, 2020.

## 2. Materials and Methods

### 2.1. Study Setting

The study was conducted in East Hararghe Zone, one of the 24 zones in Oromia region, located in eastern Ethiopia. Administratively, the zone is divided into 24 districts and four towns and has an estimated total population of 3,821,021, women in reproductive age of 844,445 and pregnant women of 132,590 in 2019 [24]. According to the zonal health office's annual report in 2019, there were five hospitals and 120 health centers, of which all hospitals and 35 health centers have trained health professionals (on postpartum family planning national guideline) and stocked with related supply commodities, providing postpartum contraceptives service in the zone [24]. The zone reported estimated 77% institutional delivery, 63% contraceptive acceptance, and 5.5 total fertility rates in 2019 [24]. The study conducted randomly selected two hospitals and eight health centers from 10 March to 09 April 2020.

### 2.2. Design, Population, and Sampling

An institution-based cross-sectional study was conducted. All women who give birth in public health facilities in East Hararghe Zone were the source population. Women who gave birth in randomly selected public health facilities during study period constituted the study population.

The sample size (*n* = 546) was computed using a single population proportion formula with the following assumptions: confidence level of 95%, margin of error of 5%, proportion of utilization of immediate postpartum LARC (21.6%) in Sidama, southern Ethiopia [21], design effect of 2, and 5% nonresponse rate. Study participants were selected using a multistage stratified sampling technique. First, we stratified facilities as hospitals (*n* = 5) and health centers (*n* = 35). Then, two hospitals and eight health centers were randomly selected using simple random sampling. Third, after estimated sample size was proportionally allocated to each selected health facility (based on the average flow in the previous six months), eligible women were included until the sample size was reached.

### 2.3. Data Collection Tool and Measurement

A pretested structured questionnaire adapted from related literature [25–27] was used to collect data. The questionnaire consists of sociodemographic and reproductive health conditions, healthcare factors, knowledge and attitude about LARC, disrespect and abuse during childbirth, and utilization of family planning. The questionnaire was first prepared in English and translated to local language (Afaan Oromoo) and back to English by two experts with good command of both languages. At discharge, ten trained diploma nurses collected the data through a face-to-face interview in a quiet private room. The overall data collection process was supervised by trained BSc nurses at each facility.

### 2.4. Data Processing and Analysis

After checking for completeness, data were entered to EpiData version 3.1 and analyzed using SPSS version 24. Descriptive statistics such as frequency and measure of central tendency/dispersion were used to characterize study participants. Before analysis, the internal consistencies of items were checked for each composite index variable using reliability analysis (Cronbach *α*). Utilization of immediate postpartum LARC was the dependent variable while sociodemographic conditions, reproductive factors, and service delivery-related information were the independent variable. Utilization of immediate postpartum LARC was defined as insertion any LARC method (IUCD or implants) within 48 hours after delivery [28]. Utilization was coded as “1” for “Yes”, and “0” for “No.” Bivariable and multivariable logistic regression analyses were fitted to identify factors associated with utilization of immediate postpartum LARC. Adjusted odds ratio (aOR) with a 95% confidence interval was used to report association, and significance was declared at *p* value < 0.05.

## 3. Results

### 3.1. Characteristics of Participants

Of total 546 immediate postpartum women invited to the study, 530 (97.1%) participants were responded/involved in the study. The mean age of participants was 26.1 (±5.4) years. Majority of the respondents were married (97.5%), gave birth vaginally (83%), and travelled more than 30 minutes to reach the nearest public health facilities (70.6%) (Table 1). One out of five (21.7%) women had experienced at least one abortion. The majority of women delivered by spontaneous vaginal delivery (83.2%) and alive birth (93.8%). Only 55 (13.5%) of women had optimal, recommendable (at least 36 months) interbirth interval time, and 14% of women did not have a plan to have a child in the next two years (Table 2).

### 3.2. Utilization of Immediate Postpartum LARC

A total of 98 (18.5%; 95% CI: 15%, 22%) women reported receiving LARC upon discharge. Majority of the women received IUCD (69.4%), with the remaining having implants. A quarter (27%) of women decided about contraceptive utilization with their partners jointly. The main reasons reported for not using were fear of the procedure (38.7%), religious restrictions (15.7%), lack of information (15.5%), desire to have more children (13.2%), and no discussion done yet with the partner (8.1%).

A total of 465 (88%) women reported that they have heard about at least one modern contraceptive method; majority reported injectables (99.4%), pills (95.1%), and implants (83.7%). The major sources of information were health extension workers (68.6%), healthcare workers (57.6%), and mass media (25.0%). Only 161 (30.4%) of all women were counselled on contraceptives. In addition, only 16% participants discussed contraceptive use with their partner. Three-fourth (74.2%) of the participants had good attitude toward LARC. A total of 408 (77.0%) participants reported facing any form of disrespect and abuse during childbirth.

### 3.3. Factors Associated with Utilization of Immediate Postpartum LARC

We found that distance from the nearest public health facility, counselling on family planning, discussion with partner about contraceptives, and disrespect and abuse during childbirth were independently associated with utilization of immediate postpartum LARC. Women who reported discussing about contraception with their partners were 6.69 times (AOR = 6.69, 95% CI: 3.54, 12.61) more likely to use immediate postpartum LARC compared to women who did not. Similarly, women who received counselling on postpartum contraceptives were 5.37 (AOR = 5.37, 95% CI: 3.00, 9.63) times more likely to use immediate postpartum LARC compared to those who did not. However, women who were residing in >30-minute foot walking distance from the nearest health facility were 53% (AOR = 0.47, 95% CI: 0.26, 0.85) less likely to use immediate postpartum LARC compared to their counterpart. Women who reported facing disrespect and abuse during childbirth were less likely to use immediate postpartum LARC (AOR = 0.22; 95% CI: 0.12, 0.40) compared to their counterpart (Table 3).

## 4. Discussion

We investigated the magnitude and associated factors of immediate postpartum LARC use among women delivered in public health facilities in East Hararghe Zone, eastern Ethiopia. We found that slightly lower than one in five women delivering in the participating facilities reported starting LARC at discharge. Discussing about contraceptives with a partner, receiving counselling, distance from the nearest health facility, and disrespect and abuse during childbirth were found to be independent predictors of LARC utilization.

Our finding is similar to the studies conducted in Sidama, southern Ethiopia (21.6%) [21] Mekele, northern Ethiopia (16.4%) [29], rural Kenya (20%) [30], and Mexico (19%) [31] and is also similar to evidence from other LMIC (15%) [17]. However, this finding is higher than the study done in Bale, southeast Ethiopia (12.4%) [22]. This might be related with the fact that we included all LARC, unlike the study in Bale, which was specific to IUCD only [22]. Besides, difference in the sociodemographic conditions or use of other contraceptive methods might contribute to the difference [32]. This finding is, however, lower than similar studies conducted in Ethiopia: Durame, southern Ethiopia (36.7%) [32]; Adaba, southeast Ethiopia (30.3%) [33]; Jimma, southwest Ethiopia (22.9%) [34]; Mizan-Aman, southwest Ethiopia (25.1%) [35]; and Harar, eastern Ethiopia (38%) [36]. This difference might be related to the differences in the population which were mainly from urban areas compared to three-fourth of our study participating coming from the rural areas. In addition, the proportion of women who were counselled on contraceptives (during antenatal care, latent phase of labour, and immediate postpartum LARC) was significantly lower in our study (30.9% versus 52.1%, 23.5% versus 62.9%, and 17.3% versus 63.2%, respectively) [32]. Moreover, the proportion of discussion with partner on contraceptive was significantly lower (16%) in our study compared to similar previous studies (16% vs. 86.3%) [36] and (16% vs. 78.2%) [33]. The counselling and discussion with partners on contraceptives might make women familiar with immediate postpartum LARC utilization.

Regarding the method-mix, 69.4% was implants and 30.6% was IUCD. This implies around two in every three women in immediate postpartum period used implants as the mean of birth control in this study. This was in line with the findings in most previous studies conducted in different parts of Ethiopia, which reported implants as the birth control for at least three-fourth of women in Durame [32], Jimma [34], Mizan-Aman [35], Harar [36], Mekele [29], Bahirdar [37], and Debra-Tabor [38]. This higher proportion of implant use might be due to convenience and privacy as implants are inserted under the skin into the upper arm area whereas IUCDs are inserted into the uterus [35]. Besides, women might think that it is painful while inserting IUCDs into the uterus especially during immediate postpartum, during sexual intercourse, or while walking [22]. Moreover, previous studies also reported that more than half (54%) of the women agreed that insertion of IUCD leads to loss of privacy [35] and one in every three (30%) women agreed that insertion of IUCD highly painful and restricts normal activities [22].

We were surprised that factors like the number of living children, having antenatal care, and length of birth interval were not associated with LARC utilization. This indicates the presence of missed opportunities in counselling women about the importance of postpartum family planning during antenatal care visits or the use of other short acting contraceptive methods, and only few intends to be pregnant in the near future; efforts should be made to support such women to use family planning to support in decreasing unmet need [39, 40].

Given that women living far from health facilities will more likely miss postnatal care, the best opportunity to start using family planning, it is worrisome that women living far away from health facilities were less likely to start LARC [41]. Mechanisms to support such women use family planning including using the health extension workers should be implemented. Although it is not our primary outcome, the fact that three-fourth of women reported facing disrespect and abuse during their childbirth—which is also found to deter LARC utilization—requires attention [38, 42].

In congruent with previous studies, women who received counselling on postpartum contraceptives were more likely to utilize LARC [21, 32]. This strengthens the importance of implementing integrated reproductive health services including counselling on family planning, during antenatal, intranatal, and postnatal visits. Similarly, women who reported discussing on contraceptives with their partners were more likely to use LARCs. Such findings were reported previously: in Debra Tabor, northwest Ethiopia [38]; Dendi, western Ethiopia [43]; and Gozamen, northwest Ethiopia [44]. In a male-dominant population of Ethiopia, having the consent of males will determine whether a woman would use or not majority of reproductive health services, including family planning.

The strengths of this study were the inclusion of hospitals and health centers which serve women with different risks of complications which hence can be generalized to eastern Ethiopia and beyond. We, however, believe the following should be considered during interpretation of our findings. Though immediate postpartum family planning is part of routine recommendations, its uptake might be affected by majority of women who might not be stable to discuss or decide its use during this stressful event. Given that majority of women would not come to facilities with partner, it may take time to obtain consent of the males, which might underestimate the number of women using postpartum contraception. Besides, the presence of high exclusive breast feeding in the settings may affect women's decision of LARC use [16, 45].

## 5. Conclusions

In conclusion, almost one in five women delivering in health facilities in eastern Ethiopia started using LARC. Provision of respectful maternity care and ensuring reproductive and sexual rights of women should be central to realize women in need of contraceptive utilizing a family planning method of their choice. Counselling on family planning should emphasize on including males who are predominantly decision-makers of household health seeking behaviour. Studying all aspects of contraceptives, unlike our focus on LARC, is required to understand the demand for contraceptives and respective met needs.

## Figures and Tables

**Table 1:** Sociodemographic characteristics of women gave birth in public health facilities in East Hararghe Zone, eastern Ethiopia, 2020 (*n* = 530).

Characteristic	Frequency	Percent
Type of health facility		
Health center	434	81.9
Hospital	96	18.1
Residence area		
Urban	128	24.2
Rural	402	75.8
Age (in years)		
15-24	177	33.5
25-34	296	55.8
≥35	67	12.7
Marital status		
Married	517	97.5
Single/divorced	13	2.5
Religion		
Muslim	471	88.9
Orthodox	44	8.3
Protestant	15	2.8
Ethnicity		
Oromo	458	86.4
Amhara	34	6.4
Guraghe	18	3.4
Other	20	3.8
Women's occupation		
Housewife	468	88.3
Merchant	16	3.0
Employee	32	6.0
Other	35	2.7
Partner's occupation		
Farmer	387	74.6
Merchant	50	9.6
Employee	60	11.6
Other	21	4.2
Education of women		
No formal education	284	53.5
Primary education	195	36.8
Secondary and above	51	9.6
Education of partner		
No formal education	203	39.3
Primary education	237	45.8
Secondary and above	77	14.9
Family size		
≤3	140	26.4
4-5	199	37.5
≥6	191	36.0
Average monthly income (in ETB)		
≤1000	193	36.4
1001-2000	207	39.1
>2000	130	24.5
Time to reach nearby public health facility		
≤30 minutes	156	29.4
>30 minutes	374	70.6

ETB: Ethiopian birr.

**Table 2:** Reproductive characteristics of women give birth in public health facilities in East Hararghe Zone, eastern Ethiopia, 2020 (*n* = 530).

Characteristic	Frequency	Percent
Gravidity		
1	115	21.7
2-4	256	48.3
>4	156	30.0
Parity		
1	124	23.4
2-4	275	51.9
>4	131	24.7
Previous abortion		
Yes	115	27.7
No	300	72.3
Number of alive children		
0-2	237	44.7
3-4	172	32.5
≥5	121	22.8
Length of inter-birth interval (months)		
<24	50	12.3
24-36	301	74.1
>36	55	13.5
Mode of delivery		
SVD	441	83.2
Instrumental	67	12.6
Cesarean delivery	22	4.2
Birth outcome (of newborn)		
Alive	497	93.8
Stillbirth	33	6.2
Gender of main birth attendant		
Male	265	49.6
Female	267	50.4
Pregnancy intention		
Wanted	415	78.3
Unwanted	115	21.7
Time to have next child		
I do not want	75	14.2
When God allows	202	38.1
Within 24 months	17	3.2
24-36 months	77	14.5
After 36 months	159	30.0.

SVD: spontaneous vaginal delivery.

**Table 3:** Factors associated with utilization of immediate postpartum LARC among women giving birth in public health facilities in East Hararghe Zone, eastern Ethiopia, 2020 (*n* = 530).

Characteristics	Utilization of LARC		cOR (95% CI)	aOR (95% CI)
	Yes, *n* (%)	No, *n* (%)		
Residence area				
Urban	49 (38.3)	79 (61.7)	4.47 (2.81, 7.11)^∗∗∗^	1.42 (0.68, 2.99)
Rural	49 (12.2)	353 (87.8)	1	1
Monthly income (in Ethiopian birrs)				
≤1000	21 (11.0)	170 (89.0)	1	1
1001-2000	42 (15.7)	225 (84.3)	1.39 (0.76, 2.52)	0.73 (0.34, 1.56)
>2000	20 (42.6)	24 (57.4)	4.64 (2.60, 8.26)^∗∗∗^	1.10 (0.46, 2.56)
Mode of delivery				
SVD	80 (18.1)	361 (81.9)	1	1
Instrumental delivery	7 (10.4)	60 (89.6)	0.53 (0.23, 1.19)	1.01 (0.36, 2.80)
Caesarian delivery	11 (50.0)	11 (50.0)	4.51 (1.89, 10.77)^∗∗∗^	3.19 (0.94, 10.86)
Time to reach nearby health facility				
>30 minutes	42 (11.2)	332 (88.8)	0.23 (0.14, 0.36)^∗∗∗^	0.47 (0.26, 0.87)^∗^
≤30 minutes	56 (35.9)	100 (64.1)	1	1
Previous use of modern contraceptives				
Yes	57 (34.1)	110 (65.9)	4.07 (2.58, 6.42)^∗∗∗^	0.94 (0.49, 1.80)
No	41 (11.0)	322 (88.7)	1	1
Antenatal care attendance				
Yes	86 (23.1)	287 (76.9)	3.62 (1.92, 6.84)^∗∗∗^	1.38 (0.63, 3.01)
No	12 (7.6)	145 (92.4)	1	1
Counseling about contraceptives				
Yes	71 (44.1)	90 (55.9)	10.00 (6.06, 16.48)^∗∗∗^	5.37 (3.00, 9.63)^∗∗∗^
No	27 (7.3)	342 (92.7)	1	1
Discussion with partner on contraceptives				
Yes	55 (64.7)	30 (35.3)	17.14 (9.94, 29.55)^∗∗∗^	6.69 (3.54,12.61)^∗∗∗^
No	43 (9.7)	402 (90.3)	1	1
Main decision maker on contraceptives				
Husband	9 (4.4)	196 (95.6)	1	1
Me	37 (18.7)	161 (81.3)	5.00 (2.35, 10.68)^∗∗∗^	1.90 (0.80, 4.49)
Both	52 (40.9)	75 (59.1)	5.10 (7.09, 32.16)^∗∗∗^	1.61 (0.59, 4.41)
Attitude toward LARC				
Good	90 (22.9)	303 (77.1)	4.79 (2.26, 10.16)^∗∗∗^	1.69 (0.46, 2.99)
Poor	8 (5.8)	129 (94.2)	1	1
Disrespect and abuse during childbirth				
Yes	42 (10.3)	366 (89.7)	0.13 (0.08, 0.22)^∗∗∗^	0.22 (0.12, 0.40)^∗∗∗^
No	56 (45.9)	66 (54.1)	1	1

^∗^ *p* < 0.05; ^∗∗^*p* < 0.01; ^∗∗∗^*p* < 0.001; LARC: long acting reversible contraceptives; SVD: spontaneous vaginal delivery; cOR: crude odds ratio; aOR: adjusted odds ratio.

## Data Availability

Data that support the findings are available from the correspondence author on reasonable request.

## Abbreviations

AOR: Adjusted odds ratio
CPR: Contraceptive prevalence rate
IUCD: Intra uterine contraceptive device
LARC: Long acting reversible contraceptive
LMIC: Low and middle income countries
WHO: World Health Organization.

## References

[1] WHO (2017). *Regional Meeting to Strengthen Capacity in the new WHO family planning guidelines: Towards universal reproductive health coverage in SDGs era*.

[2] USAID, holistic approach enhances family planning programs (2014). *Respond’s Experience with the SEED Programming Model*.

[3] Darroch J., Sedgh G., Ball H. (2011). *Contraceptive Technologies: Responding to Women’s Needs*.

[4] Singh S., Sedgh G., Hussain R. (2010). *Unintended pregnancy: worldwide levels, trends, and outcomes*.

[5] Singh S., Sedgh G., Hussain R., Eilers M. (2012). *Incidence of unintended pregnancies worldwide in 2012 and trends since 1995*.

[6] Hubacher D., Mavranezouli I., McGinn E. (2008). Unintended pregnancy in sub-Saharan Africa: magnitude of the problem and potential role of contraceptive implants to alleviate it. *Contraception*.

[7] EDHS (2016). Central Statistical Agency (CSA) [Ethiopia] and ICF. 2016. *Ethiopia Demographic and Health Survey 2016*.

[8] Fite R. O., Mohammedamin A., Abebe T. W. (2018). Unintended pregnancy and associated factors among pregnant women in Arsi Negele Woreda, West Arsi Zone, Ethiopia. *BMC Research Notes*.

[9] Kassa N., Berhane Y., Worku A. (2012). Predictors of unintended pregnancy in Kersa, Eastern Ethiopia, 2010. *Reproductive Health*.

[10] Bearak J., Popinchalk A., Ganatra B. (2020). Unintended pregnancy and abortion by income, region, and the legal status of abortion: estimates from a comprehensive model for 1990-2019. *The Lancet Global Health*.

[11] Joshi R., Khadilkar S., Patel M. (2015). Global trends in use of long-acting reversible and permanent methods of contraception: seeking a balance. *International Journal of Gynecology & Obstetrics*.

[12] WHO (2019). *Trends in maternal mortality 2000 to 2017*.

[13] UNICEF (2019). *Child Mortalty Estimate Country Specific Neonatal Mortality Rate*.

[14] Hug L., Alexander M., You D., Alkema L., UN Inter-agency Group for Child Mortality Estimation (2019). National, regional, and global levels and trends in neonatal mortality between 1990 and 2017, with scenario-based projections to 2030: a systematic analysis. *The Lancet Global Health*.

[15] WHO, World Health Organization (2013). *(WHO): Programming Strategies for Postpartum Family Planning*.

[16] Pasha O., Goudar S. S., Patel A. (2015). Postpartum contraceptive use and unmet need for family planning in five low-income countries. *Reproductive Health*.

[17] Harrison M. S., Goldenberg R. L. (2017). Immediate postpartum use of long-acting reversible contraceptives in low- and middle-income countries. *Maternal Health, Neonatology, and Perinatology*.

[18] EMDHS, Ethiopian Public Health Institute (EPHI) [Ethiopia] and ICF (2019). *2019. Ethiopia Mini Demographic and Health Survey 2019: Key Indicators*.

[19] Shabiby M. M., Karanja J. G., Odawa F. (2015). Factors influencing uptake of contraceptive implants in the immediate postpartum period among HIV infected and uninfected women at two Kenyan District hospitals. *BMC Women's Health*.

[20] Belay L., Birara M. (2018). Factors affecting long-term and permanent contraceptive uptake among immediate post-partum mothers at Saint Paul’s Hospital millennium medical college, Addis Ababa, Ethiopia: a cross-sectional study. *Ethiopian Journal of Reproductive Health (EJRH)*.

[21] Tefera L. B., Abera M., Fikru C., Tesfaye D. J. (2017). Utilization of immediate post-partum intra uterine contraceptive device and associated factors: a facility based cross sectional study among mothers delivered at public health facilities of Sidama Zone, South Ethiopia. *Journal of Pregnancy and Child Health*.

[22] Gonie A., Worku C., Assefa T., Bogale D., Girma A. (2018). Acceptability and factors associated with post-partum IUCD use among women who gave birth at Bale Zone health facilities, Southeast-Ethiopia. *Contraception and Reproductive Medicine*.

[23] Olson D. J., Piller A. (2013). Ethiopia: an emerging family planning success story. *Studies in Family Planning*.

[24] EHZD (2020). *East Hararghe Zonal Health Office’s Annual Report of 2019 (Unpublished Data)*.

[25] Teferra A. S., Wondifraw A. A. (2015). Determinants of long acting contraceptive use among reproductive age women in Ethiopia: Evidence from EDHS 2011. *Science Journal of Public Health*.

[26] Wilson S., Tennant C., Sammel M. D., Schreiber C. (2014). Immediate postpartum etonogestrel implant: a contraception option with long- term continuation. *Contraception*.

[27] Gautam R., Arya K. N., Kharakwal S., Singh S., Trivedi M. (2014). Overview of immediate PPIUCD application in Bundelkhand region. *Journal of Evolution of Medical and Dental Sciences*.

[28] ACOG, The American College of Obstetricians and Gynecologists (2016). Committee Opinion No. 670: Immediate Postpartum Long-Acting Reversible Contraception. *Contraception*.

[29] Gebremichael H. (2014). Acceptance of long acting contraceptive methods and associated factors among women in Mekelle City, Northern Ethiopia. *Science Journal of Public Health*.

[30] Ontiri S., Ndirangu G., Kabue M., Biesma R., Stekelenburg J., Ouma C. (2019). Long-acting reversible contraception uptake and associated factors among women of reproductive age in rural Kenya. *International Journal of Environmental Research and Public Health*.

[31] Potter J. E., Hubert C., White K. (2017). The availability and use of postpartum LARC in Mexico and among Hispanics in the United States. *Maternal and Child Health Journal*.

[32] Tamrie Y. E., Hanna E. G., Argaw M. D. (2015). Determinants of long acting reversible contraception method use among mothers in extended postpartum period, Durame Town, Southern Ethiopia: a cross sectional community based survey. *Health*.

[33] H F., A K., EA Y., G H., M T. (2017). Prevalence and determinant factors of long acting contraceptive utilization among married women of reproductive age in Adaba town, West Arsi Zone, Oromia, Ethiopia. *Journal of Women's Health Care*.

[34] B T., D W. (2017). Prevalence, perceptions and factors contributing to long acting reversible contraception use among family planning clients, Jimma Town, Oromiya Region, South-West Ethiopia. *Journal of Women's Health Care*.

[35] Meleko A., Sileshi S., Bekele Y. (2017). Utilization of long acting reversible contraceptive methods and its associated factors among women of reproductive age groups in Mizan–Aman Town, Bench Maji Zone, South West Ethiopia. *Journal of Women's Health Care*.

[36] Shiferaw K., Musa A. (2017). Assessment of utilization of long acting reversible contraceptive and associated factors among women of reproductive age in Harar City, Ethiopia. *Pan African medical journal*.

[37] Gelagay A. A., Koye D. N., Yeshita H. Y. (2015). Demand for long acting contraceptive methods among married HIV positive women attending care at public health facilities at Bahir Dar City, Northwest Ethiopia. *Reproductive Health*.

[38] Yalew S. A., Zeleke B. M., Teferra A. S. (2015). Demand for long acting contraceptive methods and associated factors among family planning service users, Northwest Ethiopia: *a health facility based cross sectional study*. *Biomed Central research notes*.

[39] Cheslack-Postava K., Suominen A., Jokiranta E. (2014). Increased risk of autism spectrum disorders at short and long interpregnancy intervals in Finland. *Journal of the American Academy of Child & Adolescent Psychiatry*.

[40] Gunnes N., Surén P., Bresnahan M. (2013). Interpregnancy interval and risk of autistic disorder. *Epidemiology*.

[41] Taresa A., Tura G., Alemu T. (2019). Determinants of long-acting reversible contraceptive method utilization among married women in Assosa town in Western Ethiopia: a case-control study. *Journal of Midwifery and Reproductive Health*.

[42] Kujawski S., Mbaruku G., Freedman L. P., Ramsey K., Moyo W., Kruk M. E. (2015). Association Between Disrespect and Abuse During Childbirth and Women’s Confidence in Health Facilities in Tanzania. *Maternal and Child Health Journal*.

[43] Sahilemichael A., Temesgen K. (2015). Determinants of long acting reversible contraceptives use among child bearing age women in Dendi District, Western Ethiopia. *Journal of Women's Health Care*.

[44] Gizaw W., Zewdu F., Abuhay M., Bayu H. (2017). Extended postpartum modern contraceptive utilization and associated factors among women in Gozamen district, East Gojam Zone, Northwest Ethiopia, 2014. *Insights in Reproductive Medicine*.

[45] Alemayehu M., Belachew T., Tilahun T. (2012). Factors associated with utilization of long acting and permanent contraceptive methods among married women of reproductive age in Mekelle town, Tigray region, North Ethiopia. *BMC Pregnancy and Childbirth*.

[46] CIOMS and WHO (2008). *International Ethical Guidelines for Biomedical Research Involving Human Subjects*.

